# Reliability-Based Low Fatigue Life Analysis of Turbine Blisk with Generalized Regression Extreme Neural Network Method

**DOI:** 10.3390/ma12091545

**Published:** 2019-05-10

**Authors:** Chunyi Zhang, Jingshan Wei, Huizhe Jing, Chengwei Fei, Wenzhong Tang

**Affiliations:** 1 School of Mechanical and Power Engineering, Harbin University of Science and Technology, Harbin 150080, China; zhangchunyi@hrbust.edu.cn (C.Z.); wjs19931208@163.com (J.W.); jinghuizhe@163.com (H.J.); 2 Department of Aeronautics and Astronautics, Fudan University, Shanghai 200433, China; 3 School of Computer Science and Technology, Beihang University, Beijing 10191, China; tangwenzhong@buaa.edu.cn

**Keywords:** turbine blisk, low cycle fatigue life, reliability analysis, generalized regression neural network, extremum response surface method

## Abstract

Turbine blisk low cycle fatigue (LCF) is affected by various factors such as heat load, structural load, operation parameters and material parameters; it seriously influences the reliability and performance of the blisk and aeroengine. To study the influence of thermal-structural coupling on the reliability of blisk LCF life, the generalized regression extreme neural network (GRENN) method was proposed by integrating the basic thoughts of generalized regression neural network (GRNN) and the extreme response surface method (ERSM). The mathematical model of the developed GRENN method was first established in respect of the LCF life model and the ERSM model. The method and procedure for reliability and sensitivity analysis based on the GRENN model were discussed. Next, the reliability and sensitivity analyses of blisk LCF life were performed utilizing the GRENN method under a thermal-structural interaction by regarding the randomness of gas temperature, rotation speed, material parameters, LCF performance parameters and the minimum fatigue life point of the objective of study. The analytical results reveal that the reliability degree was 0.99848 and the fatigue life is 9419 cycles for blisk LCF life when the allowable value is 6000 cycles so that the blisk has some life margin relative to 4500 cycles in the deterministic analysis. In comparison with ERSM, the computing time and precision of the proposed GRENN under 10,000 simulations is 1.311 s and 99.95%. This is improved by 15.18% in computational efficiency and 1.39% in accuracy, respectively. Moreover, high efficiency and high precision of the developed GRENN become more obvious with the increasing number of simulations. In light of the sensitivity analysis, the fatigue ductility index and temperature are the key factors of determining blisk LCF life because their effect probabilities reach 41% and 26%, respectively. Material density, rotor speed, the fatigue ductility coefficient, the fatigue strength coefficient and the fatigue ductility index are also significant parameters for LCF life. Poisson’s ratio and elastic modulus of materials have little effect. The efforts of this paper validate the feasibility and validity of GRENN in the reliability analysis of blisk LCF life and give the influence degrees of various random parameters on blisk LCF life, which are promising to provide useful insights for the probabilistic optimization of turbine blisk LCF life.

## 1. Introduction 

As a heat-end core component of an aeroengine, a turbine blisk endures complex alternating loads due to operation in a severe environment with high temperatures and high rotation speeds. In this case, it is easy to produce large plastic deformation for blisk and to induce the low cycle fatigue (LCF) failure of blisk [1,2]. Most of the parameters that significantly effect blisk LCF failure have some randomness [3]. To improve the safety and reliability of a turbine blisk to ensure the high performance of an aeroengine, it is important to study blisk LCF life reliability from a probabilistic perspective [4,5,6,7,8].

The LCF life of structures has been widely investigated. Sun et al. established a nonlinear model for LCF life of a steam turbine rotor under a temperature-stress coupling field by considering the relationship between cyclic stress and strain and validated the model to be accurate and reasonable in describing damage accumulation [9]. Letcher et al. proposed an energy-based critical fatigue life prediction approach, which derived the approximate failure cycle index from the ratio of the total accumulation of energy in the fracture process to the one-cycle strain energy [10]. Bargmann et al. discussed the full-probability quick integral algorithm based on the Coffin-Manson-Neuber local strain-fatigue theory [11]. Zhu et al. discussed the probabilistic LCF life prediction of a turbine disk under uncertainties [12,13]. Viadro et al. studied the reliability of stiffened bending plates [14]. Repetto et al. discussed the role of parameter uncertainty in the damage prediction of the alongwind-induced fatigue and long term simulation of wind-induced fatigue loadings [15,16]. Most of the above work was conducted based on numerical simulation methods (or-called direct simulation methods) with Monte Carlo (MC) simulation [15,16,17,18,19,20]. Generally, the direct simulation methods are powerful for the deterministic analyses of component LCF life. However, for the probabilistic analyses of component LCF life with thousands of iterations and MC simulations, it is unbelievable to efficiently perform blisk fatigue life analysis owing to excess computational burden (loads) and unacceptable computational efficiency; although this method has satisfactory computing precision against engineering practice. Therefore, it is urgent to seek an alternative effective method for direct methods to address this issue.

In respect of the in-depth investigation of structural fatigue probabilistic analyses, the response surface method (RSM, also called surrogate model method) is indeed an alternative method to direct simulation methods [21,22,23,24,25,26]. With the development of structural reliability theory and methods, various surrogate methods have emerged [27,28,29]. To improve the computational efficiency and accuracy of RSM for complex structural reliability analysis, Bai et al. proposed a distributed collaborative response surface method for the mechanical dynamic assemble reliability analysis of aeroengine high pressure turbine blade-tip clearance [30]. Hurtado et al. proposed a highly efficient surrogate method, a support vector machine, for structural reliability analysis with small samples [31]. Zhang et al. developed an extremum response surface method (ERSM) in respect of the extreme thought, to address the transient problem in the dynamic reliability analysis of a flexible mechanism and validated the ERSM to be precise for the reliability analysis of a flexible manipulator [32]. Lu et al. developed an improved Kriging method by integrating the Kriging algorithm and ERSM for the reliability and sensitivity analyses of a compressor blisk regarding multiple failure (deformation failure, stress failure and strain failure) modes [33]. From the efforts in References [32,33], it can be seen that ERSM has the potential to handle the transient problem in structural dynamic probabilistic analyses with a high simulation accuracy and efficiency, which provides useful insight into the process of the reliability analysis of blisk LCF life with the consideration of aeroengine operating conditions. For another, the developed ERSM does not satisfy the requirement of engineering in computing precision, derived from the weakness in processing the involved nonlinear probabilistic analyses.

With the development of neural network technology recently, the nonlinear problem was skillfully addressed by developing a generalized regression neural network (GRNN) due to the strong nonlinear mapping capability and robustness [34]. Zhao et al. established an all-purpose regression neural network model based on a freight volume condition and validated the effectiveness of this model in freight volume prediction by modeling adaptive training and extrapolation evaluation in terms of historical statistical data of freight volume and related samples and economic indicators [35]. Li et al. fused the drosophila optimization algorithm and GRNN to build the prediction model of power loads for power load prediction and this model had a strong nonlinear fitting ability [36]. Sun et al. compared the GRNN model with the back propagation neural network (BPNN) model based on air quality prediction and the GRNN method needed less training time and had better stability, a higher fitting precision as well, compared to the BPNN model [37]. Wang et al. validated the strengths of the GRNN method again by waveguide orientation [38]. Therefore, the GRNN method has been comprehensively verified to be highly computationally precise and efficient.

To effectively perform the reliability and sensitivity analyses of a turbine blsik LCF life, the generalized regression extremum neural network (GRENN) method is proposed in this paper; by integrating the transient procession ability of ERSM and the nonlinear mapping and small samples of GRNN, to collectively ensure and improve computing precision and efficiency. The reliability analysis of a turbine blisk LCF life was implemented based on the the developed GRENN, by considering random input variables of temperature, rotation speed, material parameters (density, Poisson’s ratio and elastic modulus) and fatigue performance parameters (fatigue ductility coefficient, fatigue strength coefficient and fatigue ductility index and fatigue strength index) as well as the output response of the minimum fatigue life. The developed GRENN method was validated by comparison with the MC method and ERSM. 

## 2. Basic Theory

### 2.1. Mathematical Model of Low Cycle Fatigue Life

The Mason-Coffin equation indicates the strain-fatigue life equation, which expresses the relationship between strain and the fatigue life of materials [39], i.e.,

(1) $$\frac{\Delta \varepsilon }{2}=\frac{{\sigma_{f}}^{\prime }}{E}{\left(2N_{f}\right)}^{b}+\varepsilon_{f}^{\prime }{\left(2N_{f}\right)}^{c}$$

where Δε is the total strain of specific structure; *E* indicates the elasticity modulus; $\sigma_{f}^{’}$ denotes the fatigue strength coefficient; $\varepsilon_{f}^{\prime }$ is the fatigue ductility coefficient; *b* indicates the fatigue strength exponent; *c* stands for the fatigue ductility exponent; *N_f_* expresses the LCF life. 

Considering the mean stress, *σ_m_*, inducted by complex loads during aeroengine operation, Equation (1) can be rewritten by the Morrow correction equation [40], i.e.,

(2) $$\frac{\Delta \varepsilon }{2}=\frac{\sigma_{f}^{\prime }-\sigma_{m}}{E}{\left(2N_{f}\right)}^{b}+\varepsilon_{f}^{’}{\left(2N_{f}\right)}^{c}.$$

Considering many cyclic loads, the LCF life can be classically computed by the line damage accumulation (Miner) law, i.e.,

(3) $$D=\overset{r}{\underset{i=1}{\sum }}\frac{n_{i}}{N_{i}}$$

in which *D* indicates the fatigue damage; *r* is the number of loading levels; *n_i_* denotes the cyclic number under the *i*th loading level; *N_i_* is the fatigue life corresponding to the *i*th loading level. 

### 2.2. Mathematical Model of Extremum Response Surface Method 

To effectively process the transient problem in the dynamic reliability analysis of blisk LCF life involving nonlinear and transient features of numerous parameters, i.e., gas temperature, rotation speed, material parameters (density, Posion’s ratio, elasticity modulus) and fatigue performance parameters (fatigue strength coefficient, fatigue ductility coefficient, fatigue strength exponent and fatigue ductility exponent), the ERSM proposed in Reference [32] was adapted by simplifying the response process of the LCF life as an extreme value (maximum value or minimum value) in an analytical time domain. When ***X*** and *y_e_* were used to indicate the input parameters set and the output extremum response, the ERSM model *y_e_*(***X***) of the dynamic system [32] can be written as

(4) $$y_{e}\left(X\right)=f\left(X\right)=\left\{y_{e}^{\left(j\right)}\left(X^{\left(j\right)}\right)\right\}$$

where $X^{\left(j\right)}$ is the *j*th group of the input samples; $y_{e}^{\left(j\right)}\left(X^{\left(j\right)}\right)$ indicates the output extremum response during a time domain. 

In previous studies, most of the ERSM models were built based on polynomials [33] and these models are usually inefficient in model fitting because the polynomials are unworkable for highly nonlinear problems and the large computing burden (requiring a large number of samples for modeling) required for blisk fatigue life probabilistic analysis. Thus, the GRNN method, with a strong nonlinear mapping ability and robustness, was applied by combining ERSM in this paper to address the issues of modeling precision and efficiency, which result from nonlinearity and large samples.

### 2.3. Mathematical Model of Generated Regression Extremum Neural Network Method 

**Figure 1:**
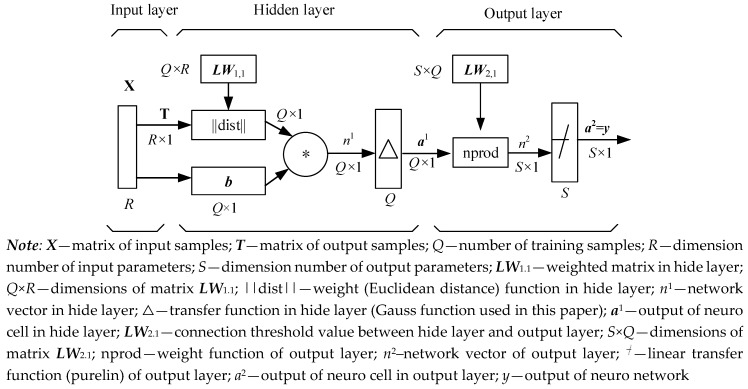
Schematic diagram of generalized regression extreme neural network (GRENN) method.

GRNN is a feedforward neural network model based on nonlinear regression theory, including input layer (first layer), hidden layer (middle layer) and output layer (last layer), as shown in Figure 1. 

By inputting train samples into the input layer, the input matrix ***X*** and output matrix (denoted by ***T***) are expressed by

(5) $$X=\left[\begin{array}{cccc} x_{11} & x_{12} & \cdots & x_{1Q}\\ x_{21} & x_{22} & \cdots & x_{2Q}\\ \cdots & \cdots & \cdots & \cdots \\ x_{R1} & x_{R2} & \cdots & x_{RQ} \end{array}\right],\;T=\left[\begin{array}{cccc} t_{11} & t_{12} & \cdots & t_{1Q}\\ t_{21} & t_{22} & \cdots & t_{2Q}\\ \cdots & \cdots & \cdots & \cdots \\ t_{S1} & t_{S2} & \cdots & t_{SQ} \end{array}\right]$$

where *x_ji_* (*i* = 1,2, …, *Q*; *j* = 1,2, …, *R*) is the *j*th input sample in the *i*th group of training samples; *t_ji_* indicates the *j*th output sample in the *i*th group of training samples; *R* is the number of input variables; *S* is the number of output variables; *Q* is the number of samples in the training set.

The number of neurons in the hidden layer was equal to the number of samples in the training set, the layer weight function was the Euclidean distance function (expressed in ||dist||), and the implicit layer weight matrix is calculated as follows:

(6) $$LW_{1,1}=X^{T}$$

The threshold for *Q* hidden layer neural units is ***b***:

(7) $$b={\left[b_{1},b_{2},\cdots b_{Q}\right]}^{T}$$

in which $b_{1}=b_{1}=\cdots =b_{Q}=\frac{0.8326}{\sigma }$, *σ* is the smooth factor of the Gauss function.

The transfer function of the hidden layer is usually based on the Gaussian radial basis function. The number of neurons in the hidden layer *Q* is equal to the number of training samples and each neuron corresponds to one training sample. When the weight matrix and threshold value of the hidden layer neural unit were determined, the output $a_{i}^{j}$ of the ith hidden layer neuron is

(8) $$a_{i}^{j}=exp(-\frac{0.8326}{\sigma }\|LW_{1.i}-x_{j}\|{}^{2}),\;j=1,2,\cdots ,Q\;;i=1,2,\cdots ,Q$$

in which $LW_{1.i}={\left[x_{h1},x_{h2},\cdots ,x_{hR}\right]}^{T}$ (h=1,2, ⋯,Q) is the vector of the ith implicit layer weight matrix **LW**_1,1_; $x_{j}={\left[x_{j1},x_{j2},\cdots ,x_{jR}\right]}^{T}$ is the vector of the jth training samples. let $a^{j}=\left[a_{1}^{j},a_{2}^{j},\cdots ,a_{i}^{j},\cdots a_{Q}^{j}\right],$ which is the output vector of Q nerve cells corresponding to the jth group of input samples.

Regarding the connection weight ***LW***_2,1_ between the hidden layer and output layer as the output matrix of the training set of samples, which is denoted by ***T***, i.e.,

(9) $$LW_{2,1}=T.$$

The output layer is the third layer of GRNN. Based on GRNN Equations (8) and (9), vector *n^j^* can be computed by

(10) $$n^{j}=\frac{LW_{2,1}{\left[a^{j}\right]}^{T}}{\sum_{i=1}^{Q}a_{i}^{j}}.$$

With regard to the line transfer function$\;y^{j}=purelin\left(n^{j}\right)$ of *n^j^*, the mathematical model of GRNN for the response of the *j*th group of training samples is expressed by

(11) $$y^{j}=purelin\left(n^{j}\right)=\frac{LW_{2,1}{\left[a^{j}\right]}^{T}}{\sum_{i=1}^{Q}a_{i}^{j}}$$

where *exp* is a natural exponential function.

With respect to the format in Equation (4), the mathematical model of GRENN is

(12) $$y_{min}^{j}=Min\left\{\frac{LW_{2,1}{\left[a^{j}\right]}^{T}}{\sum_{i=1}^{Q}a_{i}^{j}}\right\}.$$

### 2.4. Reliability Sensitivity Analyses Approaches with GRENN Model

Assuming that $y^{*}$ is the allowable LCF life and $y_{min}^{j}$ is the performance function of the structural fatigue life, the limit state function of LCF life is derived as [24]

(13) $$Z=y_{min}^{j}-y^{*}.$$

In Equation (13), *Z* > 0 indicates that the blisk structure is secure, while *Z* < 0 reveals a failure. When random input variables are independently mutual, their means and variance are denoted by $\mu =\left[\mu_{1},\mu_{2}\cdots \mu_{n}\right]$ and $D=\left[D_{1},D_{2}\cdots D_{n}\right]$, respectively, we can gain [18]

(14) $$\{\begin{array}{c} E\left(Z\right)=\mu_{Z}\left(\mu_{1},\mu_{2},\cdots ,\mu_{n};D_{1},D_{2},\cdots ,D_{n}\right)\\ D\left(Z\right)=D_{Z}\left(\mu_{1},\mu_{2},\cdots ,\mu_{n};D_{1},D_{2},\cdots ,D_{n}\right) \end{array}$$

in which E(Z) is mean function and D(Z) is variance function.

When the limit state function of the structural LCF life (Equation (13)) obeys a normal distribution, the reliability degree *P_r_* is expressed as [25]

(15) $$P_{r}=\Phi \left(\frac{\mu_{Z}}{\sqrt{D_{Z}}}\right)$$

where *µ_z_* is the mean matrix of a limit state function *Z*; *D_z_* is the variance matrix of a limit state function.

The sensitivity reflects the level of sensitivity of the input random variables on the failure probability of a structural system response, which is promising to determine the extent to which these parameters effect the response and then provide a useful guide for structural design and optimization [41]. 

With the proposed GRENN method, the sensitivity degree can be determined by the mean matrix ***µ*** and variance ***D*** of input random variables [42], i.e.,

(16) $$\frac{\partial P_{r}}{\partial \mu^{T}}=\frac{\partial P_{r}}{\partial \left(\frac{\mu_{Z}}{\sqrt{D_{Z}}}\right)}\left(\frac{\partial \left(\frac{\mu_{Z}}{\sqrt{D_{Z}}}\right)}{\partial \mu_{Z}}\frac{\partial \mu_{Z}}{\partial \mu^{T}}+\frac{\partial \left(\frac{\mu_{Z}}{\sqrt{D_{Z}}}\right)}{\partial D_{Z}}\frac{\partial \mu_{Z}}{\partial \mu^{T}}\right);\;\frac{\partial P_{r}}{\partial D^{T}}=\frac{\partial P_{r}}{\partial \left(\frac{\mu_{Z}}{\sqrt{D_{Z}}}\right)}\left(\frac{\partial \left(\frac{\mu_{Z}}{\sqrt{D_{Z}}}\right)}{\partial \mu_{Z}}\frac{\partial \mu_{Z}}{\partial D^{T}}+\frac{\partial \left(\frac{\mu_{Z}}{\sqrt{D_{Z}}}\right)}{\partial D_{Z}}\frac{\partial \mu_{Z}}{\partial D^{T}}\right)$$

in which

(17) $$\{\begin{array}{c} \frac{\partial P_{r}}{\partial \left(\mu_{Z}/\sqrt{D_{Z}}\right)}=P_{r},\frac{\partial \left(\mu_{Z}/\sqrt{D_{Z}}\right)}{\partial \mu_{Z}}=\frac{1}{\sqrt{D_{Z}}},\frac{\partial \left(\mu_{Z}/\sqrt{D_{Z}}\right)}{\partial D_{Z}}=-\frac{\mu_{Z}}{2}D_{Z}^{-\frac{3}{2}}\\ \frac{\partial \mu_{Z}}{\partial \mu^{T}}={\left[\frac{\partial \mu_{Z}}{\partial \mu_{1}},\frac{\partial \mu_{Z}}{\partial \mu_{2}},\cdots ,\frac{\partial \mu_{Z}}{\partial \mu_{n}}\right]}^{T}\\ \frac{\partial \mu_{Z}}{\partial D^{T}}={\left[\frac{\partial \mu_{Z}}{\partial D_{1}},\frac{\partial \mu_{Z}}{\partial D_{2}},\cdots ,\frac{\partial \mu_{Z}}{\partial D_{n}}\right]}^{T}\\ \frac{\partial D_{Z}}{\partial \mu^{T}}={\left[\frac{\partial D_{Z}}{\partial \mu_{1}},\frac{\partial D_{Z}}{\partial \mu_{1}},\cdots ,\frac{\partial D_{Z}}{\partial \mu_{1}}\right]}^{T}\\ \frac{\partial D_{Z}}{\partial D^{T}}={\left[\frac{\partial D_{Z}}{\partial D_{1}},\frac{\partial D_{Z}}{\partial D_{1}},\cdots ,\frac{\partial D_{Z}}{\partial D_{1}}\right]}^{T} \end{array}.$$

**Figure 2:**
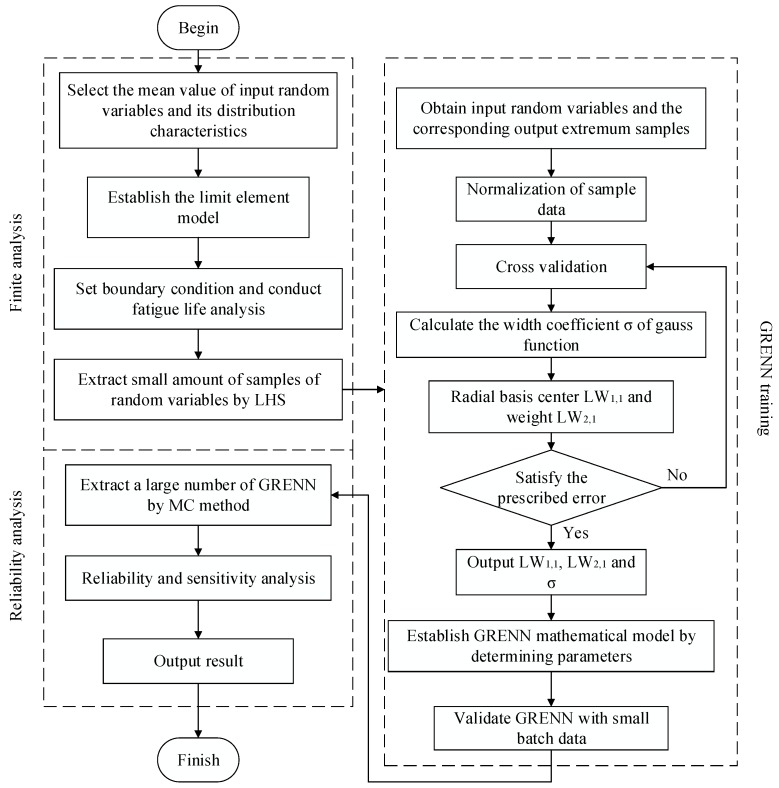
Flow chart of reliability analysis with GRENN method.

In respect of the GRENN method and thermal-structure coupling, the flowchart of the blisk LCF life reliability analysis is drawn in Figure 2 and its basic procedure is described below.

***Step 1:***  Build the finite element (FE) model of blisk in a workbench environment;
***Step 2:***  Consider the means of the input random variables (i.e., gas temperature, rotation speed, material parameters and fatigue performance parameters) and set boundary conditions to conduct the blisk FE analysis under the interaction of heat load, centrifugal load and then gain the minimum fatigue point as the design point of the blisk reliability design.
***Step 3:***  Extract small samples of the input random variables using the Latin hypercube sampling (LHS) method and perform FE analyses for each group of samples to gain the output responses (blisk LCF life) and extract the minimum values of the responses as a training sample set by combining the input samples.
***Step 4:***  Training the GRENN model by computing the optimal smooth factors, radial basis function and connection weights with the cross validation method [26], through the normalization of training samples.
***Step 5:***  Structure of the limit state function of blisk LCF life with the established GRENN model.
***Step 6:***  Check the precision of the GRENN model. If unacceptable, return to ***Step 4***; if acceptable, conduct ***Step 7***.
***Step 7:***  Calculate the reliability degree and sensitivity degree of the fatigue life and input variables, by conducting the reliability and sensitivity analyses of blisk LCF life with thermal-structure coupling, through a large number of samples extracted by the MC method.

## 3. Reliability and Sensitivity Analyses of Blisk Low Cycle Fatigue Life

### 3.1. Random Variables Selection

In this study, we selected the high-pressure turbine blisk of an aeroengine with the high-temperature GH4133 as the object of study. In fact, the uncertainty and randomness of some parameters are the basic nature in blisk LCF life design and prediction [15]. By comprehensively regarding the engineering practice, the exiting data and the basic properties of parameter uncertainty studied by Repetto, et al. [15], the probabilistic analysis of the blisk LCF life was performed by the randomness of numerous reasonably-selected parameters, such as rotation speed *ω*, gas temperature *T*, material density *ρ*, heat conductivity *λ*, elasticity modulus *E*, fatigue strength efficient *σ′_f_*, fatigue ductility coefficient *ε′_f_*, fatigue strength index *b* and fatigue ductility index *c*. To simplify the calculation by combining engineering practices [43] and the present data, the selected variables were summed to be independent mutually and obey normal distributions. The distributions of the variables are listed in Table 1. 

### 3.2. Deterministic Analysis of Blisk Low Cycle Fatigue Life 

**Figure 3:**
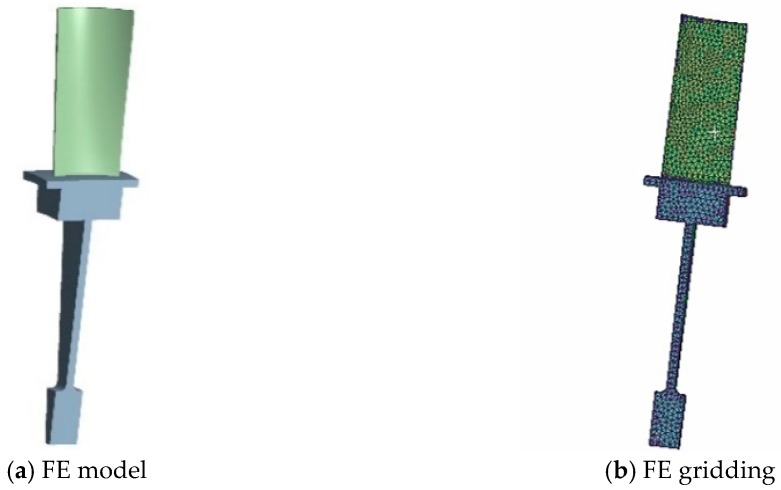
FE model and gridding of a turbine blisk.

For the static analysis of the blisk, the blisk stress inducted by aerodynamic loads can be ignored because it is far less than that caused by the centrifugal load and heat load [43]. The deterministic analysis of the blisk was completed by regarding the interaction of temperature and centrifugal loads, under a workbench 16.0 environment in the computer with a central processing unit (CPU) mode of Xeon E5-2630V3 (Intel Corporation, Santa Clara, CA, USA) and RAM (Intel Corporation) of 64 GB. Due to the symmetry of the blisk, we selected 1/40 of the whole blisk for analysis to reduce the computational burden [44]. The FE models are shown in Figure 3, with 31,380 nodes and 17,111 elements. The thermodynamic analysis of the blisk was implemented in which the heat energy of a high temperature gas is transferred to the surface of the blisk according to the heat conduction law and heat convection. In light of thermodynamic theory, the temperature distribution on the blisk surface can be calculated by the empirical formula, i.e.,

(18) $$T=T_{a}+(T_{a}-T_{b})\left(\frac{R^{m}-R_{a}^{m}}{R_{b}^{m}-R_{a}^{m}}\right)$$

in which $T_{a}$ is the temperature at blisk-root; $R_{a}$ is the radius of blisk-root edge;$\;T_{b}$ is the temperature at blisk-tip;$\;R_{b}$ is the radius of blisk-tip; R is the radius of blisk in a different position; *m* = 2 was determined for the high temperature alloy GH4133B [39].

**Figure 4:**
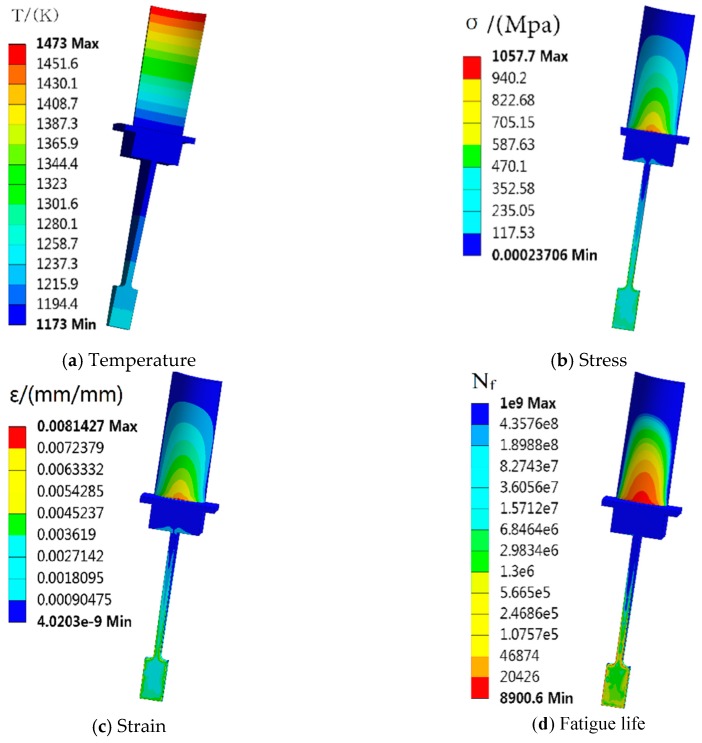
Nephgrams of the responses of blisk stress and fatigue life.

By the displacement constraint of the blisk’s inner diameter to restrict the degrees of freedom in the directions *x*, *y* and *z*, the deterministic analysis of the blisk was finished based on the means of the input variables in Table 1. The distributions of temperature, equivalent stress and equivalent strain are shown in Figure 4a–c. As seen in Figure 4a–c, the maximum stress of the blisk was 1 057.7 Mpa on the blade-root and the minimum strain was 8.142 7 × 10^−3^ m/m. Therefore, the node of the maximum strain on the blade-root was selected as the object of study for the blisk LCF life analysis. In terms of the Mason-Coffin formula in Equation (2) and the Miner line accumulative damage rule in Equation (3), the fatigue life values at the max-strain point of the blisk areshown in Figure 4d. It is illustrated in Figure 4d that the minimum fatigue life was 8900.6 cycles. In respect of the double safety coefficients in engineering, the LCF of the blisk should be about 4450 cycles based on the deterministic analysis. 

### 3.3. Low Cycle Fatigue Life Models of Blisk with GRENN Method

With regard to the distribution of the input random variables in Table 1, 150 samples (a small batch) were extracted by LHS technology. Based on these samples and FE analyses, the corresponding output responses (minimum LCF lives) were computed as the samples together with the extracted input samples. One hundred and twenty groups of samples were selected from the pool of training samples as training samples and the remain 30 groups of samples were selected as the test samples for the GRENN model. 

**Figure 5:**
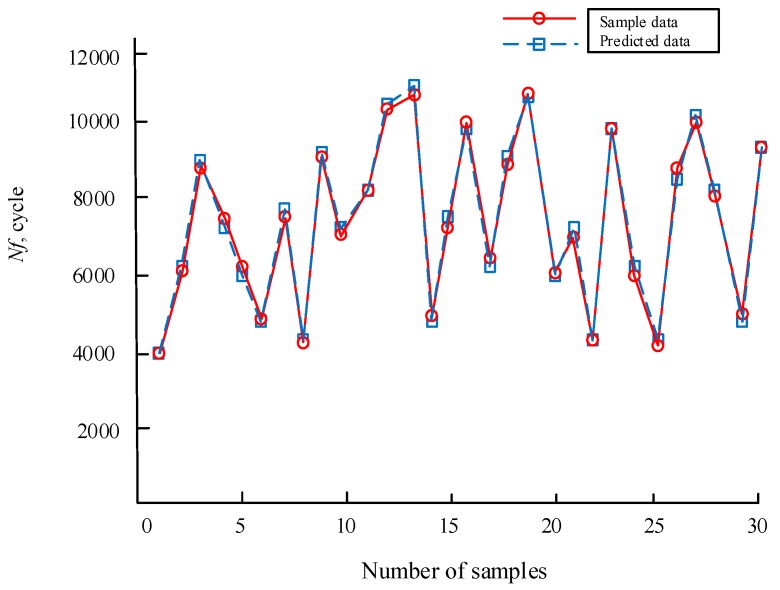
Predicted results of the GRENN model with 30 groups of samples.

Regarding the Gauss function as a transfer function in the hidden layer, the implicit layer weight ***LW***_1.1_ of the hidden layer was computed using the Euclidean distance method. The outputs of GRENN training were taken as the connection weights ***LW***_2,1_ between the hidden layer and the output layer. The original samples data should be normalized for each parameter. The normalized data were adopted to train the GRENN and then to gain the parameters of GRENN (the implicit layer weight ***LW***_1.1_ of the hidden layer, the connection weights ***LW***_2,1_ and the smooth factor *σ*) by the cross validation method [35], in which ***b*** and ***LW***_1.1_ and ***LW***_1,2_ (computed by Equations (6), (7) and (9)) are summarized in Equation(19). By inputting the values of these parameters into Equation (12), the GRENN model can be gained. The remaining 30 groups of samples were employed to test the established GRENN model. The prediction results are shown in Figure 5. From Figure 5, it can be seen that the predicted data were almost consistent with the true sample data, which indicates a high prediction precision for the developed GRENN model.

(19) $$\{\begin{array}{cc} LW_{1,1} & ={\left[\begin{array}{cccccc} -0.4189 & 0.0946 & \cdots & 0.9054 & 0.9459 & 0.7973\\ -0.8912 & -0.5646 & \cdots & 0.7415 & -0.2517 & -0.1020\\ 1.0000 & -0.5646 & \cdots & -0.7415 & 0.7959 & -0.5510\\ -0.7852 & -0.7315 & \cdots & -0.8792 & -0.1678 & 0.4765\\ 0.2245 & -0.8639 & \cdots & -0.2789 & -0.6190 & -0.3878\\ 0.5839 & 0.3020 & \cdots & 0.2483 & 1.0000 & -0.7315\\ 0.6510 & 0.2752 & \cdots & -0.6644 & 0.1678 & -0.5973\\ -0.6892 & 0.8514 & \cdots & 0.6216 & -0.9865 & -0.4865\\ -0.0470 & -0.5302 & \cdots & 0.7584 & 0.6107 & 0.0336\\ -0.1757 & -0.8108 & \cdots & 0.7432 & 0.2838 & -0.3919 \end{array}\right]}_{10\times 120}^{T}\\ LW_{2,1} & ={\left[\begin{array}{cccccc} \;-0.9061 & -0.8701 & \cdots & \;-0.9627 & -0.9968 & -0.9400 \end{array}\right]}_{1\times 120}\\ b & ={[2.8710\;2.8710\;\cdots \;2.8710\;2.8710\;2.8710]}_{1\times 120}^{T} \end{array}$$

### 3.4. Reliability Analysis of Blisk Low Cycle Fatigue Life with GRENN Model

**Figure 6:**
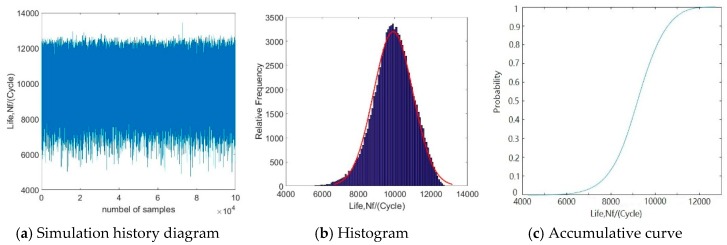
Reliability analysis results of blisk fatigue life with the GRENN method.

In this subsection, the reliability analysis of the blisk LCF life with the GRENN model was performed by 10,000 simulations with the MC method. The historical simulation diagram and histogram of the blisk LCF life are shown in Figure 6. As shown in Figure 6, the blisk minimum fatigue life followed a normal distribution with a mean of 9419 cycles and a standard deviation 967 cycles. As the allowable fatigue life y^*^ = 6 000 cycles, the reliability degree *P_r_* of the blisk LCF life was 0.99848 in line with Equations (13) and (15). The gained reliability degree basically catered for blisk design in engineering. In this case, the obtained fatigue life of a blisk was 6000 cycles in respect to the reliability analysis. However, the minimum LCF life of the deterministic analysis was 8900.6 cycles, as shown Figure 4d. In respect to the double safe coefficients, the safe fatigue life of blisk design was about 4450 cycles in engineering in line with the deterministic analysis. Therefore, it is revealed that the deterministic analysis method is backward-looking relative to ~4450 cycles of the probabilistic analysis method for blisk LCF life prediction at 6000 cycles, because 4450 cycles was far less than 6000 cycles. 

### 3.5. Sensitivity Analysis of Blisk Low Cycle Fatigue Life with GRENN Method

**Figure 7:**
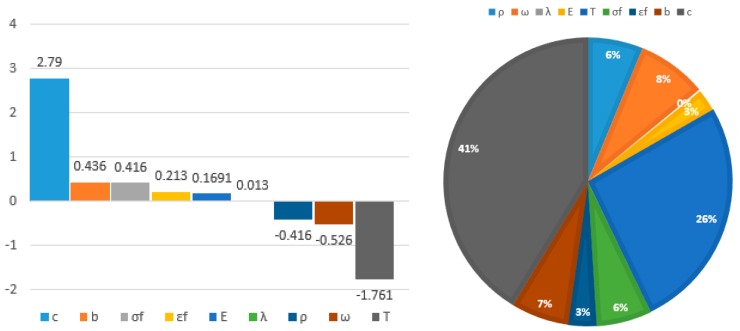
Sensitivity degree distributions of random parameters on blisk LCF life.

Sensitivity reflects the level of sensitivity of the input random variables on blisk reliability, which is helpful to find the major impact factors and then guide structural design. Sensitivity involves the sensitivity degree and the effect probability. The sensitivity degree is defined by the effect of the input parameters on the output response with positive and negative signs. A positive sign indicates the input parameter was positively correlated with the output response and vice versa for a negative sign. Effect probability is defined as the ratio of the sensitivity degree of one input parameter to the total sensitivity degree of all input parameters. In terms of Equations (12)–(17), the sensitivity results are listed in Table 2 and Figure 7.

As demonstrated in Table 2 and Figure 7, the fatigue ductility index *c* and gas temperature *T* were two major influencing parameters because their effect probabilities and sensitivity degree were 41.3% and 0.27929, as well as 26.16% and −0.176122, respectively. Other parameters play small effects on blisk reliability. Therefore, *T* and *c* should be considered and controlled in blisk design with priority.

### 3.6. Validation of GRENN

To validate the effectiveness and validation of the GRENN method, the MC method and ERSM were used in the reliability analyses of blisk LCF life under different simulations based on the same computation conditions and random variables. The computing time and reliability degrees are presented in Table 3 and Table 4. In Table 3 and Table 4, the precision for the method *D_p_* was computed under 10,000 simulations, by

(20) $$D_{p}=1-\frac{\left|\gamma_{a}-\gamma_{m}\right|}{\gamma_{a}}\times 100\%$$

in which *γ_a_* is the reliability degree of the MC method; *γ_m_* indicates the reliability degree of ERSM or the GRENN method. 

As shown in Table 3, with the increasing number of simulations, the computing time increases for the MC method, ERSM and the GRENN method. For the MC method, the simulations larger than 10,000 require an excessive computational burden so that the MC method is unworkable for such large simulations. However, ERSM and the GRENN method only take a few seconds and thus can breezily implement simulations from 100 to 1,000,000. Relative to ERSM, the developed GRENN method spends less time and is highly computationally efficient, and that the strength of the GRENN method becomes more obvious with an increase in the number of simulations. For instance, under 10,000 simulations, the GRENN method reduces the computing time by 0.048 s and improves the computational efficiency by 3.843% relative to the ERSM, while the simulation time is reduced by 2.138 s and the efficiency is improved by 50.42%. Therefore, it is revealed that the proposed GRENN method has a strong computing power and is highly computationally efficient in probabilistic simulations. Meanwhile, the potential of high efficiency becomes stronger and more simulations are required. 

In Table 4, the reliability degree computed by the MC method under 10,000 simulations is regarded as a reference. In this case, we find that the reliability degrees of ERSM and the GRENN method were 0.9824 and 0.9923 under 10,000 simulations, and their computational precisions were 98.56% and 99.95% so that the GRENN method improves the precision by 1.39%. Additionally, with an increasing number of simulations, the reliability degree of a turbine blisk increases and the developed GRENN method is more accurate than ERSM. 

Therefore, the developed GRENN method is highly computationally precise and efficient and the strengths become more obvious for more simulations.

## 4. Conclusions

The aim of this paper was to propose a new reliability analysis method, i.e., the generalized regression extreme neural network (GRENN) method, for the reliability analysis of blisk LCF life, to improve the life and performance of turbine blisks. The developed GRENN absorbed the strengths of a generalized regression neural network (GRNN) in nonlinear mapping and small sample-based modeling, and the extremum response surface method (ERSM) for handling the transient problem of the dynamic reliability analysis of blisk LCF life. Through this study, some conclusions are summarized as follows:

(1) The reliability degree of blisk LCF life was 0.99848 when the life allowable value was 6000 cycles. Relative to 4450 cycles acquired from the deterministic analysis after considering the double coefficient of a safe life, the LCF (6000 cycles to ensure a reliability degree of 0.99848) of the blisk obtained from the reliability design had enough life margin (about 1550 cycles) to ensure the operation of the blisk structure.
(2) From the sensitivity analysis of a blisk, the fatigue ductility index *c* and gas temperature *T* played key roles in blisk LCF life evaluation and design. *T* and *c* were positively and negatively correlated with blisk life, respectively. The conclusions can significantly guide the optimization and design of blisk LCF life.
(3) Through the comparison of the methods, it is demonstrated that the developed GRENN method is far better than ERSM in modeling precision and computing efficiency and is basically consistent with the MC method. Moreover, the strengths of the GRENN method become more obvious with the increasing number of simulations. It is fully supported that the proposed GRENN method is a high-accuracy and high-efficiency method to address the key questions of nonlinearity, transients and large sample-based modeling.

In summary, the efforts of this paper provide a promising method (GRENN method) for the nonlinear dynamic reliability analysis of complex structures and enrich and develop mechanical reliability theory. 

## Figures and Tables

**Table 1:** Distributions of random variables.

Random Variables	Mean *μ*	Standard Deviation *δ*	Distribution
Density *ρ, kg.m*^−3^	8210	328.4	Normal
Rotate speed *ω*, *rad·s*^−*1*^	1168	35	Normal
Heat conductivity *λ*, *W·m*^−1^·°C^−1^	23	0.005	Normal
Modulus of elasticity, *E*, *MPa*	163000	4890	Normal
Blade-root temperature *T_a_* , *k*	1173.15	35.2	Normal
Blade-tip temperature *T_b_*_,_ *k*	1473.15	47	Normal
Fatigue strength efficient *σ′_f_*	1419	42.5	Normal
Fatigue ductility coefficient *ε′_f_*	50.5	1.53	Normal
Fatigue strength index *b*	−0.1	0.005	Normal
Fatigue ductility index *c*	−0.84	0.042	Normal

**Table 2:** Sensitivity degree and impact probability of the random input parameters.

Random Parameters	Sensitivity Degree, ×10^−3^	Effect Probability, %
*ρ*	−0.41586	6.18
*ω*	−0.52565	7.81
λ	+0.0132	0.20
*E*	+0.16948	2.52
*T*	−1.76022	26.16
*σ′_f_*	+0.41615	6.18
*ε′_f_*	+0.21311	3.17
b	+0.43585	6.48
c	+2.7929	41.30

**Table 3:** Computing time of the MC method, ERSM and GRENN.

Number of Samples	Computing Time under Different Simulations, s			Reduced Time, s	Improved Efficiency, %
	MC Method	ERSM	GRENN		
10^2^	5400	1.249	1.201	0.048	3.843
10^3^	14400	1.266	1.201	0.065	5.134
10^4^	432000	1.681	1.311	0.370	15.18
10^5^	—	2.437	1.342	1.095	44.93
10^6^	—	4.312	2.138	2.174	50.42

**Table 4:** Computational precision of the reliability analysis methods under different simulations.

Samples	Reliability Degree			Precision/%		Improved Precision/%
	MC Method	ERSM	GRENN	ERSM	GRENN	
10^2^	0.85	0.76	0.79	76.24	79.25	3.01
10^3^	0.976	0.947	0.968	95.00	97.11	2.11
10^4^	0.9968	0.9824	0.9973	98.56	99.95	1.39
10^5^	—	0.98181	0.99848	98.49	99.83	1.34
10^6^	—	0.98262	0.99587	98.58	99.91	1.33
